# Future perspective on chronic kidney disease management

**DOI:** 10.1016/j.jatmed.2025.06.002

**Published:** 2025-07-24

**Authors:** Xiaomei Qiu, Xueqin Ding, Henry Liu, Qifang Li

**Affiliations:** a Department of Anesthesiology, Chengdu First People's Hospital, Chengdu 610000, China; b Department of Anesthesiology, MetroHealth Medical Center, Case Western Reserve University, Cleveland, OH 44109, USA; c Anesthesiology and Critical Care, Perelman School of Medicine, University of Pennsylvania, Philadelphia, PA 19104, USA; d Department of Anesthesiology and Perioperative Medicine, Shanghai Fourth People's Hospital, School of Medicine, Tongji University, Shanghai 200434, China

**Keywords:** Chronic kidney disease, Renal denervation, Thoracic epidural blockade, Sympathetic activity

## Abstract

Over-activation of the sympathetic nervous system is one of the major characteristics in chronic kidney disease (CKD), with renal ischemia playing a pivotal role in its development. While various pharmacologic strategies have been introduced to suppress sympathetic activity and slow the CKD progression, these approaches are often hindered by challenges such as limited effectiveness, drug resistance, adverse effects, and poor patient adherence. Renal denervation has emerged as a potential alternative that directly targets sympathetic nerve activity, and numerous studies have highlighted its promise. However, the outcomes of renal denervation remain variable, and the procedure carries the risk of disrupting the regulatory functions of the renal nervous system. Thoracic epidural blockade presents a novel therapeutic option for CKD management by modulating sympathetic nervous system activity. This review attempts to explore the pathophysiology of CKD, the involvement of the sympathetic nervous system in disease progression, the limitations of current pharmacologic treatments and renal denervation, and the potential benefits of thoracic epidural blockade in managing CKD.

## Introduction

Chronic kidney disease (CKD) is defined by structural or functional abnormalities of the kidney persisting for more than three months, significantly impacting patients’ overall health and quality of life. As of 2017, the global prevalence of CKD was 9.1 %, affecting approximately 697.5 million people across all stages of the disease.^1^ In China, the prevalence is even higher, with 10.8 % of adults over 18 diagnosed, equating to 132 million individuals.^2^ The aging population and rising prevalence of hypertension and diabetes have contributed to the steady increase in CKD cases globally. By 2021, CKD was responsible for 44.5 million disability-adjusted life years (DALYs) worldwide, underscoring its significant burden.^3^

Despite its global impact, CKD currently remains incurable, and available treatments are focused on delaying disease progression and managing complications. According to the 2024 Clinical Practice Guidelines for CKD, comprehensive treatment strategies include self-management and supportive therapies. However, even with these interventions, CKD often progresses. For instance, after CKD reaches stage G3, 3–5 % of patients progress to renal failure within five years; at stage G4, 20 % progress within two years; and at stage G5, 40 % progress within two years.^3^

The etiology of CKD varies geographically.^4^ In Western countries, secondary causes such as diabetes and hypertension dominate. In contrast, in China, primary glomerulonephritis, particularly IgA nephropathy, remains the leading cause, followed by diabetic nephropathy, hypertensive nephropathy, lupus nephritis, obstructive nephropathy, and polycystic kidney disease. Regardless of the cause, CKD involves chronic inflammation, fibrosis, parenchymal cell loss, and decreased renal regenerative capacity, leading to irreversible loss of nephrons, end-stage renal disease (ESRD), and premature death. Current therapies are limited in their effectiveness, emphasizing the urgent need for innovative treatments to prevent or reverse CKD progression.^5^

### Sympathetic nervous system and CKD

The kidneys are densely innervated by renal afferent sensory and efferent sympathetic nerves, which interact with the central nervous system. Renal sensory nerves transmit signals to the dorsal horn of the spinal cord and further to brain regions such as the subfornical organ (SFO), paraventricular nucleus (PVN), and rostral ventrolateral medulla (RVLM). These brain centers modulate sympathetic outflow via preganglionic and postganglionic neurons.^6^, ^7^, ^8^ The progression of chronic kidney disease (CKD) is closely associated with over-activation of the sympathetic nervous system (SNS).^9^, ^10^, ^11^ This heightened renal sympathetic activity results in increased renin secretion, reduced renal blood flow, and enhanced tubular sodium reabsorption.^6^, ^12^, ^13^ Norepinephrine, a principal mediator of the SNS, plays a critical role in various renal physiological processes, including regulation of renal blood flow, glomerular filtration rate, water and sodium reabsorption, and renin release.^6^, ^7^, ^12^, ^14^

Norepinephrine released from sympathetic nerve terminals works in conjunction with other substances such as Adenosine triphosphate and neuropeptide Y to regulate renal function.^15^ Elevated plasma norepinephrine levels are strongly linked to kidney injury progression.^16^

Norepinephrine acts on beta (1)-adrenergic receptors (β1-ARs) to stimulate renin production and release, while jointly influencing beta (2)-adrenergic receptors (β2-ARs) to regulate renal blood flow, glomerular filtration rate, sodium and water reabsorption, and acid-base balance. The resultant increase in renin leads to elevated angiotensin-II levels, the primary effector of the renin-angiotensin system (RAS) in hypertensive patients. In turn, Angiotensin-II amplifies sympathetic excitation by promoting norepinephrine release and inhibiting norepinephrine reuptake at adrenergic nerve terminals, further exacerbating sympathetic activity in the heart, kidneys, and vasculature.^6^ Overall, the overactivation of the renal SNS and elevated norepinephrine levels are the central drivers in the pathogenesis of CKD.

### Limitations of current pharmacologic strategies

Guidelines recommend initiating renin-angiotensin system inhibitors in patients with CKD and severe proteinuria (G1-G4, A3). Sodium-glucose cotransporter 2 inhibitors (SGLT2i), widely used in diabetic CKD, regulate global sympathetic outflow by acting on renal afferents. These agents promote glucosuria and urinary sodium excretion, reduce fluid retention, alleviate sympathetic drive, decrease cardiac load, and improve cardiorenal function.^17^ Mineralocorticoid receptor antagonists offer additional benefits by reducing proteinuria, slowing CKD progression, and suppressing fibrosis and inflammatory pathways.^18^ Additionally, a nationwide cohort study suggests that adding diuretics rather than calcium channel blockers to renin-angiotensin system inhibitors therapy may improve renal outcomes in patients with advanced CKD without compromising cardioprotection.^19^

Despite these advancements, the global incidence of CKD continues to rise. Limitations of drug therapy are well-documented, including inefficacy in some patients, resistance to treatment, adverse effects, and poor patient compliance. For example, Angiotensin-converting enzyme inhibitors (ACEi) and angiotensin receptor blockers (ARB), cornerstone therapies for CKD, have been linked to worsening renal failure, particularly in elderly hypertensive patients aged 65 and older.^20^ A study showed that discontinuing ACEi/ARB may improve renal function and enhance clinical outcomes without increasing cardiovascular risk.^21^ RAS blockade is suitable for proteinuria or hypertensive nephropathy, but cannot prevent CKD progression.^22^ Mineralocorticoid receptor blockers also exhibit anti-proteinuria effects, but whether they delay CKD progression remains to be confirmed.^23^

There is currently no therapy that can reverse the transformation of CKD.^5^ The persistent challenges associated with pharmacologic therapies underscore the urgent need for innovative strategies to address CKD. As the limitations of current approaches become increasingly evident, the development and rigorous evaluation of novel interventions are critical to improving outcomes for CKD patients.

### Renal denervation for chronic kidney disease

Renal denervation (RDN) offers a novel approach to directly modulate the SNS. By simultaneously disrupting renal afferent input to the central nervous system (CNS) and efferent output from the CNS to the kidney, RDN inhibits sympathetic outflow, leading to potential benefits for hypertension, CKD, and heart failure. Some clinical evidence indicates that RDN is safe, effective, and provides sustained blood pressure reduction in CKD patients. For instance, RDN has been shown to improve blood pressure control in CKD patients at stages G3–G4 and slow the progression of renal dysfunction.^24^, ^25^, ^26^ In hypertensive CKD sheep models, RDN resulted in sustained decreases in blood pressure and proteinuria, as well as improved GFR after 30 months of treatment.^27^

However, RDN remains controversial. Some studies report no significant changes in renal function within nine months post-procedure, and in patients with refractory hypertension, the reduction in systolic blood pressure was comparable between RDN and control groups.^28^

These discrepancies highlight several challenges. Firstly, the presence of accessory renal arteries in at least 30 % of individuals complicates complete renal denervation and may contribute to variability in antihypertensive efficacy.^29^ Secondly, nerve regeneration after RDN poses another challenge. Studies in sheep model have shown that functional and anatomical nerve regeneration is largely complete by 5.5 months post-RDN, with no discernible differences in sympathetic nerve activity compared to controls at 11 months.^30^ Finally, concerns persist regarding the potential adverse effects of permanently disrupting sympathetic nerve regulation permanently. While no significant complications have been reported, this aspect warrants further investigation to explore reversible blockade of sympathetic nerve for CKD management.

### Thoracic epidural blockade for chronic kidney disease

Thoracic epidural blockade (TEB) offers another therapeutic approach by targeting renal afferent sensory nerves that transmit signals to the CNS via the spinal cord. By blocking sympathetic outflow and sensory afferents, TEB may reduce sympathetic activation and its deleterious effects in CKD. Moreover, TEB is a reversible blockade technique and more extensively used.

### Mechanisms of thoracic epidural blockade in CKD

#### TEB may improve CKD by interrupting sympathetic afferent and efferent signaling from the heart and kidneys

CKD is not just a renal issue; it is intricately linked with hypertension and cardiovascular disease. Dysfunction in one organ, whether acute or chronic, can precipitate dysfunction in the other. For instance, renal impairment is prevalent in patients with chronic cardiovascular conditions, particularly congestive heart failure (CHF), affecting approximately 40–60 % of patients.^31^ Acute kidney injury is also a common complication in acute decompensated heart failure, occurring in roughly 24–45 % of cases. Furthermore, among all CKD patients, about 50 % of deaths are attributed to cardiovascular disease.^32^ The coexistence of CHF and renal dysfunction is a significant predictor of poor clinical outcomes.

A central mechanism linking CKD, cardiovascular disease, and hypertension is the over-activity of the sympathetic nervous system (SNS), which promotes and exacerbates these conditions.^8^, ^33^ Renal ischemia-reperfusion injury in CKD models has been shown to increase sympathetic outflow not only in the kidneys but also in the heart. Similarly, myocardial ischemia-reperfusion injury elevates sympathetic discharge affecting both the heart and kidneys, further underscoring the reciprocal relationship between these organs.^8^

The interaction between the heart and kidney is facilitated by sympathetic chains at the spinal level. Sensory nerve signals from these organs converge in the brain via the spinal cord, with the heart playing a pivotal role in regulating SNS activity. TEB therapy represents a promising remote intervention for central autonomic modulation by targeting renal afferent and efferent sympathetic nerves. By inhibiting renal sympathetic activity, epidural block disrupts maladaptive neurohormonal activation, resets CNS integration, and reduces global sympathetic efferent output. This leads to cardioprotective and vasculoprotective effects in CKD.

**Fig. 1:**
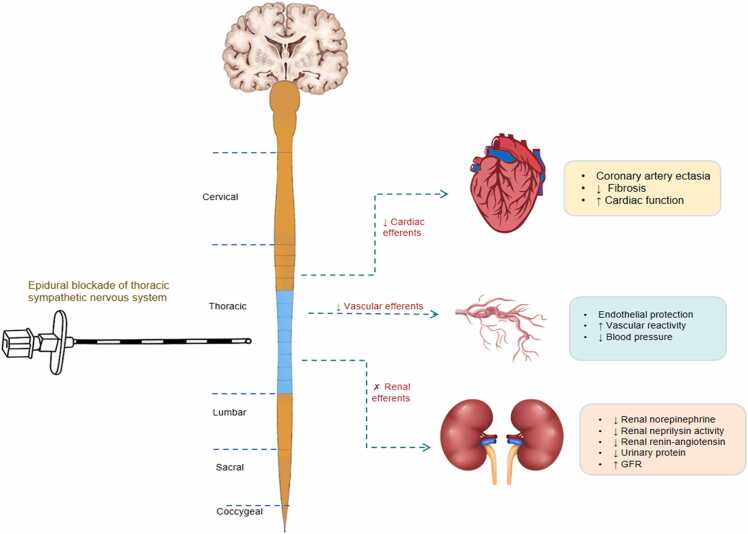
The proposed mechanisms of thoracic epidural blockade for the treatment of CKD.

Specifically, TEB interrupts sympathetic afferent and efferent signaling from the heart and kidneys. This reduces sympathetic excitation linked to hypertension and breaks the pathological interactions between these organs, providing therapeutic benefits for CHF, CKD, and hypertension (Fig. 1).

The interaction between the heart and kidney is facilitated by sympathetic chains at the spinal level. Sensory nerve signals from these organs converge in the brain via the spinal cord, with the heart playing a pivotal role in regulating SNS activity. By reversibly inhibiting renal sympathetic activity, epidural block disrupts maladaptive neurohormonal activation, resets CNS integration, and reduces global sympathetic efferent output. This leads to cardioprotective and vasculoprotective effects in CKD. This reduces sympathetic excitation linked to hypertension and breaks the pathological interactions between these organs, providing therapeutic benefits for CKD.

#### Anti-inflammatory effects of thoracic epidural block

Chronic low-grade inflammation contributes to reduced quality of life, morbidity, and premature mortality in CKD.^34^ Emerging evidence emphasizes the role of gut health in systemic inflammation.^35^, ^36^ TEB enhances peripheral and visceral vascular perfusion, improving intestinal mucosal blood flow,^37^, ^38^ reducing serum IL-6 levels,^39^ and protecting the gut from microvascular damage and leukocyte-endothelial interactions.^40^

Local anesthetics such as lidocaine and bupivacaine, commonly used in epidural blocks, exhibit dose-dependent anti-inflammatory properties. These include reducing leukocyte adhesion and migration, phagocytosis, oxidative stress, and inflammatory mediator synthesis. They also inhibit the release of pro-inflammatory cytokines like IL-1, TNF-α, and IL-8.^41^, ^42^ Lidocaine, in particular, may exert indirect systemic anti-inflammatory effects through absorption during epidural administration. However, ropivacaine appears to have minimal anti-inflammatory and bactericidal activity.

While the precise mechanisms underlying these effects remain to be elucidated, ongoing research is shedding light on this innovative strategy and its potential to be applied in CKD management.

### Current evidence of thoracic epidural block on CKD

There are many data suggesting regional anesthesia to reduce the risk of intraoperative incident, particularly in patients with CKD.^43^, ^44^ Our group is the first to investigate the impact of TEB on CKD. In an animal study, we demonstrated that epidural block with lidocaine improved renal function, mitigated renal fibrosis, and partially or fully reversed abnormalities in both renal and cardiovascular parameters.^45^ Based on this, we evaluated the effect of TEB in a patient with CKD. The patient received an epidural block with 0.8 % lidocaine administered in rapid bolus injections of 6 mL, with a lockout interval of 180 min, for 16 h daily. After seven months of treatment, the patient exhibited stable blood pressure, allowing discontinuation of antihypertensive medications during the study period. Kidney function showed notable improvement, with estimated GFR increasing from 56.18 mL/min/1.73 m² to 72.9 mL/min/1.73 m² and serum creatinine levels decreasing from 128.9 μmol/L to 103.2 μmol/L. Additionally, proteinuria reduced by over 80 % from baseline, and hematuria resolved completely.^46^ Epidural block/anesthesia is an important means of pain control during operation throughout the world, and the cost is relatively low compared to RDN, about one-third of RDN currently. Additional advantages of epidural block over RDN are summarized in Table 1.

**Table 1:** Key differences between renal denervation and thoracic epidural blockade.

Category	Renal denervation	Thoracic epidural blockade
Block range	Limited to renal nerve	Extensive, from heart to kidney
Reversibility	Damage nerve, not reversible	Reversible
Renal/nerve anatomical variations	Influence the outcome	No influence
Procedure availability	Depends on operator and equipment	Routine procedure in clinical anesthesia setting

Although TEB shows promise as a therapeutic option for CKD, its efficacy requires validation through large-scale, prospective, randomized controlled trials with extended follow-up periods. Further research is essential to explore its effects across different CKD stages and to clarify the underlying cellular and histological mechanisms. Continued investigation could position TEB as a novel and effective treatment modality for CKD management.

## Conclusions

CKD is a complex clinical syndrome leading to significant morbidity, mortality, and economic burden. Despite advancements in medical therapies, no guideline-based interventions have proven capable of halting or reversing CKD progression, particularly in ESRD. Current therapies primarily target symptoms, with limited efficacy in reversing disease progression. As discussed above RDN has been introduced as a novel therapy to treat drug resistant hypertension. It is thought to be another approach to directly modulate the sympathetic nervous system. However, RDN interrupts both the afferent inputs from the kidney to the CNS and the efferent outputs from the CNS to the kidney, resulting in suppression of sympathetic outflow and eliciting beneficial effects in both hypertension and CKD.

TEB emerges as a promising candidate in this therapeutic landscape. With its ability to induce a wide, reversible blockade of sympathetic afferents and efferents at the spinal cord level, it offers a targeted and precise means of modulating sympathetic hyperactivity—a key factor in CKD progression. By directly influencing the SNS, TEB interrupts the deleterious interactions between the heart, kidneys, and vascular system, thereby mitigating hypertension, reducing proteinuria, and improving GFR.

Nevertheless, considering the limitations of this review-such as the limited clinical evidence for thoracic epidural blockade, lack of randomized controlled trials, and potential selection bias in the cited literature, it could be concluded that multiple well-designed, controlled and randomized studies are needed to validate the potential benefits of thoracic epidural blockade in managing CKD.

In summary, TEB represents a potential new modality in the management of CKD. By targeting the autonomic nervous system's maladaptive responses, it offers a novel and integrative strategy to address the CKD progression. While promising, the path forward necessitates robust research to unlock its full therapeutic potential and refine the details of the TEB therapy.

## CRediT authorship contribution statement

**Qifang Li:** Writing – review & editing. **Henry Liu:** Writing – review & editing. **Xueqin Ding:** Writing – review & editing. **Xiaomei Qiu:** Writing – original draft. All authors have read and agreed to the published version of the manuscript.

## Consent for publication

Not applicable.

## Ethical statement

Not applicable.

## Funding

This research received no external funding.

## Declaration of competing interest

The authors declare that they have no known competing financial interests or personal relationships that could have appeared to influence the work reported in this paper. Henry Liu is an Editor-in-Chief for this journal and was not involved in the editorial review or the decision to publish this article.

## Data Availability

All data cited in this review are publicly available through the original publications listed in the references. Source data for figures are provided with this paper.
